# Clinical and social determinants of treatment outcomes in central nervous system tuberculosis in a high-resource setting: a retrospective cohort study

**DOI:** 10.1186/s12879-026-13439-8

**Published:** 2026-04-28

**Authors:** Carlos Hernandez Castillo, Meg Wilson, Laura Romo-Timme, Cassandra Recchioni, Janice Louie, Felicia C. Chow

**Affiliations:** 1 Institute for Global Health Sciences, University of California, San Francisco, San Francisco, USA; 2 Yale University School of Medicine, New Haven, USA; 3 Weill Institute for Neurosciences, Department of Neurology, University of California, San Francisco, Box 0896, 2540 23rd Street, Floor 5 (5216), San Francisco, CA 94110 USA; 4 Tuberculosis Clinic, San Francisco Department of Public Health, San Francisco, USA; 5 UCSF Center for Tuberculosis, University of California, San Francisco, San Francisco, USA; 6 Department of Medicine (Infectious Diseases), University of California, San Francisco, San Francisco, USA

**Keywords:** Tuberculous meningitis, CNS tuberculosis, Social determinants of health, Mortality, Disability, Functional impairment, cognitive impairment

## Abstract

**Background:**

Limited data are available regarding clinical and social determinants influencing treatment outcomes among patients with central nervous system (CNS) tuberculosis (TB) in high-resource settings.

**Methods:**

We performed a retrospective cohort study of patients with CNS TB from January 2011 to June 2022 at the San Francisco Department of Public Health TB Clinic and the University of California, San Francisco Medical Center. Data were abstracted from cases of confirmed or suspected CNS TB treated with anti-TB therapy. Logistic regression models were used to identify sociodemographic and clinical factors associated with mortality and functional and cognitive impairment among survivors.

**Results:**

Of 54 cases of CNS TB, 26% were microbiologically-confirmed. 31% resided in the top 3 deciles of social disadvantage. Over a mean follow-up of 10.2 months, cumulative mortality was 22%. Among survivors, 40% exhibited functional impairment and 38% demonstrated moderate to severe cognitive dysfunction. In adjusted models, having public insurance (aOR 14.94, 95% CI 1.58-141.29, *p* = 0.018) or residing in a highly deprived neighborhood (aOR 7.66, 95% CI 1.46–40.22, *p* = 0.016) was associated with functional and cognitive impairment, respectively. Additional risk factors for adverse clinical outcomes included older age, diabetes mellitus, severe disease at presentation (e.g., altered mentation, requiring intubation) and hydrocephalus.

**Conclusions:**

In this high-resource setting, cumulative mortality and disability rates among patients treated for CNS TB were comparable to outcomes reported in studies from low and middle-income countries where TB is endemic. Although limited by the modest sample size, this study demonstrates a potential link between social disadvantage and poor CNS TB outcomes, highlighting persistent inequities in TB care even within a well-resourced health system. If these findings are confirmed in a larger study, implementing a comprehensive approach that addresses the social determinants of health affecting individuals at risk for TB will be essential to improving outcomes in this devastating infection.

**Supplementary Information:**

The online version contains supplementary material available at 10.1186/s12879-026-13439-8.

## Introduction

In 2023, tuberculosis (TB) reclaimed the dubious distinction of being the leading cause of death from a single infectious disease worldwide [1]. Central nervous system (CNS) TB, the deadliest and most disabling form of the infection, accounts for approximately 1% to 2% of all TB cases and 4% to 7% of cases among people living with HIV [2–6]. One challenge to understanding the true burden of CNS TB lies in the difficulty in diagnosing the infection, in large part due to the insensitivity of microbiological testing, including microscopy and mycobacterial culture, in this paucibacillary condition [3, 7]. Although diagnostic radiologic criteria have been proposed to supplement microbiological methods, neuroimaging findings are often non-specific [7, 8].

Mortality rates are high in CNS TB, ranging from 20% to over 40% in certain populations, including in people living with HIV [3, 9, 10]. Survivors may experience a variety of long-term physical, neurologic, cognitive, and functional impairments [11–13]. In two recent systematic reviews, the prevalence of neurologic sequelae in adults with TB meningitis (TBM) was cited as 29% and 51%, although little information was available regarding the manner or severity of disability [14, 15]. While post-TBM sequelae can have a substantial impact on daily activities and quality of life, few studies have characterized functional outcomes in detail or over a longer period of follow up [10, 11].

Most studies of treatment outcomes after CNS TB are from low and middle-income countries (LMIC) where the burden of TB is high [10]. Scant data are available on characteristics and clinical outcomes of patients with CNS TB in the U.S. and other high-income settings in which the incidence of TB is low [16]. One study using administrative claims data from hospitals in California, New York, and Florida found that 55% of patients with TBM experienced a major neurological complication or death [17]. Another study documented neurologic disability at 12 months in 33% of adults with TBM cared for between 2001 and 2017 at a single tertiary care facility in the United Kingdom [18]. Although TB disproportionately affects communities living in poverty, a paucity of studies has evaluated the impact of social determinants of health on outcomes in CNS TB [19, 20], especially in high-resource settings like the U.S.

This study characterized cases of CNS TB, which included tuberculous meningitis, intracranial tuberculoma, spinal tuberculous arachnoiditis, or a combination of these syndromes, managed at the San Francisco Department of Public Health TB Clinic and the University of California, San Francisco (UCSF) Medical Center. The objectives were to describe mortality rates and functional and cognitive outcomes among patients with CNS TB in a non-endemic, high-income urban setting in the U.S. and to identify sociodemographic and clinical factors associated with these outcomes.

## Methods

### Study population

We performed a retrospective cohort study of pediatric and adult patients evaluated and treated for CNS TB between January 1, 2011 and June 1, 2022 at either (1) the San Francisco Department of Public Health TB Clinic (referred to as “TB Clinic”), where residents of San Francisco diagnosed with TB are seen in follow-up, or (2) the UCSF Medical Center, an academic tertiary and quaternary care referral center that also serves as a community hospital for surrounding neighborhoods.

The study population was drawn from two pools of patients. First, all patients with CNS TB seen for a minimum of one visit at the San Francisco Department of Public Health TB Clinic were included. Second, we queried the UCSF Medical Center’s electronic medical record system using the following broad search criteria to capture potential cases of microbiologically-confirmed or suspected CNS TB: (1) an International Classification of Diseases, Ninth or Tenth Revision (ICD-9 or ICD-10), code for CNS TB (ICD-9 013.00, 013.01, 013.10, 013.14, 013.20, 013.24, 013.30, 013.50, 013.60, 013.64, 013.80, 013.86, 013.90, 013.91, 013.94; ICD-10 A17.0, A17.1, A17.81, A17.82, A17.83, A17.89, A17.9); or (2) a cerebrospinal fluid (CSF) test result suggestive of CNS TB [e.g., positive *Mycobacterium tuberculosis* nucleic acid amplification test, positive acid-fast bacilli (AFB) culture or smear, or positive adenosine deaminase]; or (3) an ICD code for pulmonary or extrapulmonary TB coupled with a CSF white blood cell count of > 5 cells/mm3; or (4) an ICD code for meningitis or encephalitis combined with concomitant use of anti-TB medications.

Paper-based records from the San Francisco Department of Public Health TB Clinic and electronic medical records from the UCSF Medical Center were reviewed to identify patients with confirmed or suspected CNS TB treated with anti-TB therapy. Cases were excluded if insufficient information was available to establish a diagnosis of CNS or to determine whether anti-TB treatment was initiated. Identified CNS TB cases were classified as definite, probable, or possible using a uniform case definition developed to standardize diagnostic criteria for research [21]. All clinical criteria required for the definition (e.g., duration of symptoms, presence of individual signs and symptoms) were from initial presentation or from available notes closest to initial presentation; if the presence or absence of a sign or symptom was not specifically documented in the notes, it was considered to be absent.

### Data abstraction

For all confirmed or suspected cases of CNS TB, sociodemographic characteristics, risk factors for TB, co-occurring conditions, and health-related behaviors were abstracted from the medical record. In addition to insurance status at diagnosis, residential addresses at diagnosis were used to calculate the University of Wisconsin Area Deprivation Index (ADI) [22, 23], which measures neighborhood-level social disadvantage. We dichotomized ADI, a common approach in analyses that use ADI and similar tools to capture social determinants of health on a population level [24]. The top three ADI deciles (deciles 8–10) for California, representing the most severe deprivation, were compared with the lower seven deciles (deciles 1–7). Self-reported alcohol use was categorized into 3 groups (3 drinks or fewer per week; between 4 and 7 drinks per week for women and between 4 and 14 drinks for men; and more than 7 drinks per week for women and more than 14 drinks per week for men) based on a scale adopted from the Centers for Disease Control and Prevention [25]. Cigarette smoking and marijuana use were categorized as current, past, or never. Presenting signs and symptoms, TB treatment history, brain and spine imaging findings, and laboratory results from blood and CSF, including TB diagnostic evaluations, were also recorded.

### Functional and cognitive outcomes

In addition to mortality, data were abstracted by C.H.C. on cognitive and functional outcomes at the last available CNS TB-related healthcare visit using a standardized abstraction tool. Independence in activities of daily living (ADLs) was assessed using the Katz Index of Independence in ADLs, which assigns one point for independence in each of six areas: bathing, dressing, toileting, transferring, continence, and feeding [26]. Functional impairment was defined as having a score of 4 or lower, reflecting dependence in at least 2 ADLs. Cognitive impairment was assessed based on occupational therapy and other clinical notes. The cognitive domains assessed included language and verbal skills, executive functioning, memory, attention and concentration, sensation, perception, and motor skills. Patients were categorized as having “no or slight limitations” if they had no cognitive sequelae in any domain or had persistent cognitive symptoms in one domain with minimal impact on function. Patients with “moderate to severe limitations” had impairments in more than one cognitive domain but were able to perform most ADLs or had major impairment in one or more cognitive domains and required significant assistance for most ADLs.

### Statistical analysis

Sociodemographic and clinical characteristics at the time of presentation or at the first available visit were summarized using descriptive statistics and compared by outcome status (i.e., survival, functional impairment, moderate to severe limitations in cognition) using t-tests, Wilcoxon rank-sum tests, and chi-square or Fisher’s exact tests, as appropriate. For variables that differed significantly by outcome status, we used a series of logistic regression models to identify factors associated with mortality, functional impairment and cognitive impairment. Due to the modest sample size, models were first adjusted for age, sex, and race/ethnicity and then for age, sex, race/ethnicity, followed by additional adjustment for altered mental status at presentation, a proxy for disease severity that has been strongly associated with clinical outcomes in TBM [3, 12, 27]. Two patients with missing data on mental status were excluded from this model. HIV status was not included as a covariate in models due to its lack of association with clinical outcomes. A third model was developed with adjustment for neighborhood-level deprivation, as measured by the ADI. In sensitivity analyses, we repeated the models after excluding cases that could not be classified using the uniform case definition or those classified as possible cases. Statistical significance was defined as P values less than 0.05.

## Results

**Fig. 1:**
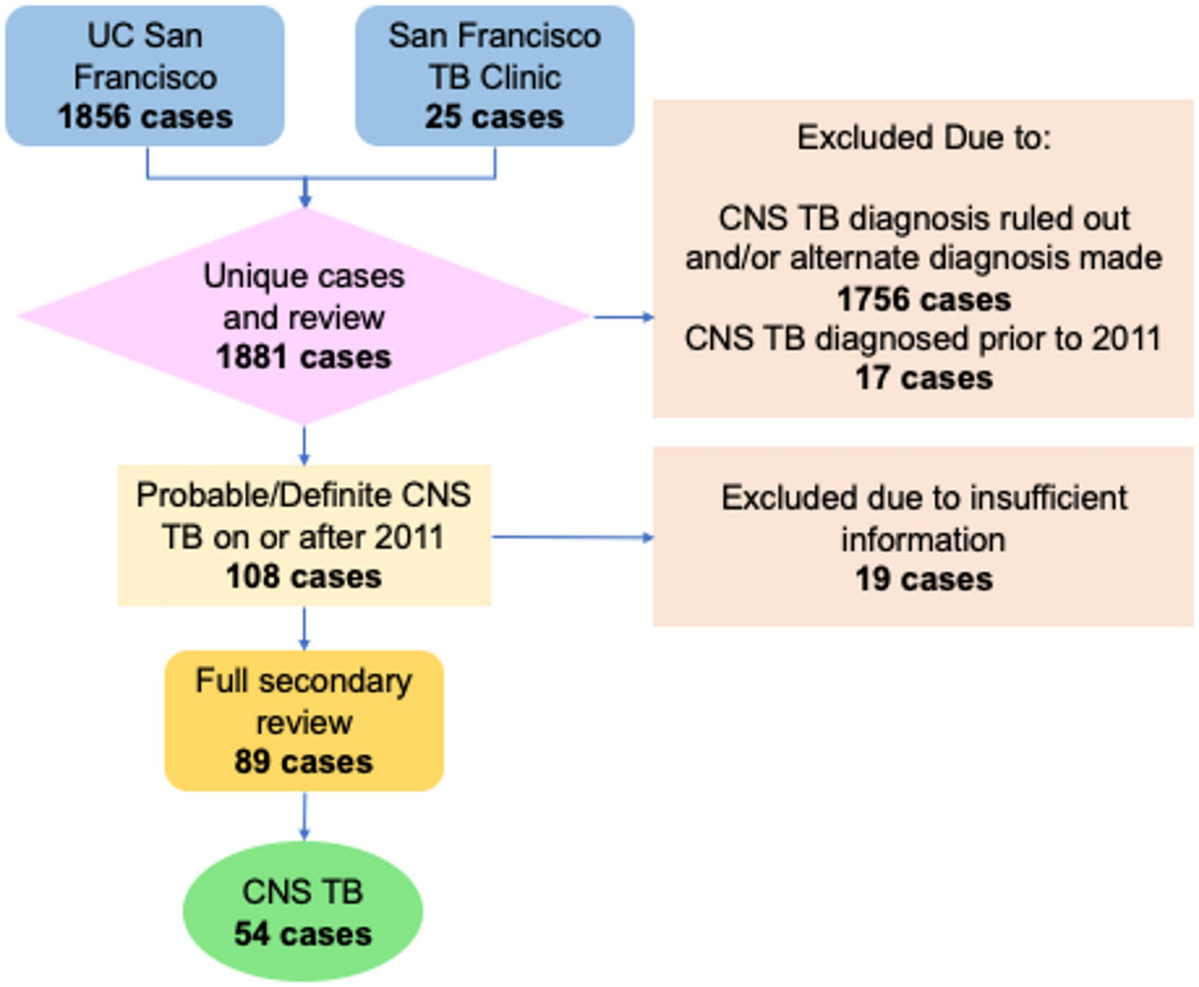
Flowchart of case identification and medical record review process

The search criteria generated a total of 1,881 potential cases for review (Fig. 1). Following preliminary assessment, 1,756 cases were excluded as non-CNS TB cases, 17 were diagnosed prior to January 1, 2011, and 19 had insufficient clinical information. A detailed review of the remaining 89 cases identified 54 patients with confirmed or suspected CNS TB. Most patients were men between 18 and 64 years. The largest racial group was Asian (41%) followed by White (28%). Hispanic or Latino individuals comprised 22% of the cohort. A majority (63%) were born outside the US, and 37% required a translator during clinical visits. Among foreign-born patients, the most common countries of origin were Mexico, the Philippines, and China. The mean time in the U.S. for individuals who were foreign-born was 21.6 years [standard deviation (SD) 17.1]. Two-thirds of patients had public insurance; 31% resided in areas within the top 3 deciles of deprivation, as measured by the ADI. Additional sociodemographic characteristics are summarized in Table 1.

**Fig. 2:**
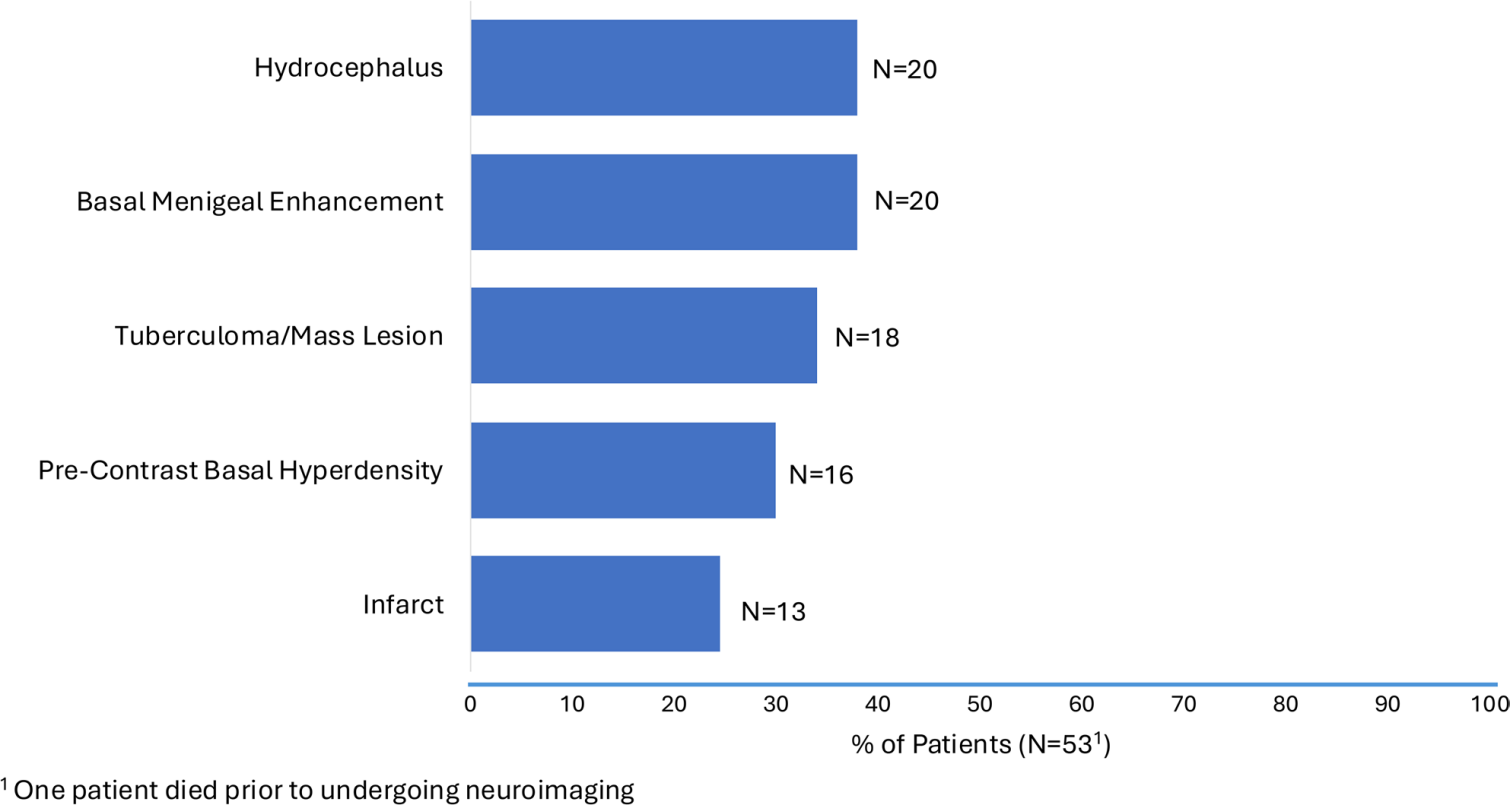
Neuroimaging findings in patients with CNS TB

As indicated in Table 1, most patients were not living with HIV infection. Among people living with HIV, all except one was on antiretroviral therapy (ART). Hypertension and diabetes mellitus were prevalent comorbidities, affecting 39% and 24% of patients, respectively. Fever (72%), focal weakness (70%), headache (61%), and altered mental status (56%) were common signs at the time of presentation (Supplemental Table 1). 30% of patients required intubation at the time of presentation. Hydrocephalus and meningeal enhancement, each observed in 37% of patients, were the most common radiologic findings (Fig. 2). CSF laboratory results at presentation or early in the diagnostic work-up were available for 47 patients. Of these, 39 (83%) had a CSF pleocytosis, of which 31 (66%) had a lymphocytic predominance, 26 (56%) had protein levels above 100 mg/dL, and 22 (47%) had hypoglycorrhachia.

**Table 1:** Clinical and sociodemographic factors of patients with CNS TB

Characteristic, *n* (%) unless noted	All patients (*N* = 54)
Age (years), mean (SD)	47 (24)
Sex at birth	
Male	30 (56)
Female	24 (44)
Race and Ethnicity	
Non-Hispanic Asian	22 (41)
Non-Hispanic White	15 (28)
Hispanic/Latino	12 (22)
Non-Hispanic Black	5 (9)
US-born	
No	34 (63)
Yes	19 (35)
Unknown	1 (2)
Non-US Country of origin	
Mexico	8 (24)
Philippines	5 (15)
China	4 (12)
Other^1^	17 (59)
Used a translator	
Yes	20 (37)
No	34 (63)
Insurance status	
Public	36 (67)
Private	12 (22)
Uninsured	2 (4)
Unknown	4 (7)
Housing status	
Unhoused or unstably housed	2 (4)
Stably housed	52 (96)
Area Deprivation Index	
≤ 7 (bottom 7 deciles)	37 (69)
≥ 8 (top 3 deciles)	17 (31)
Living with HIV infection	
Yes	6 (11)
No	45 (83)
Unknown	3 (6)
History of hypertension	
Yes	21 (39)
No	33 (61)
History of diabetes	
Yes	13 (24)
No	39 (72)
Unknown	2 (4)
History of end-stage renal disease	
Yes	2 (4)
No	52 (96)
Alcohol use	
> 14 drinks per week (men) or > 7 drinks per week (women)	4 (7.5)
4–14 drinks per week (men) or 4–7 drinks per week (women)	4 (7.5)
3 or fewer drinks per week	39 (72)
Unknown	7 (13)
Smoking	
Current Smoker	8 (15)
Former Smoker	9 (17)
Never Smoker	30 (56)
Unknown	7 (13)
Marijuana use (current/prior)	
Yes	6 (11)
No	48 (89)

^1^2 patients from Germany, Guatemala, Nicaragua, Thailand, and Vietnam, respectively, and 1 patient from India, Burma, Tunisia, Ethiopia, Soviet Union, Laos, and Pakistan, respectively

Among patients with CNS TB, 14 (26%) had microbiologically-confirmed disease based on a positive AFB culture of CSF or a brain biopsy specimen. Of these culture-positive cases, 6 (43%) had resistance to at least one first-line anti-TB drug (monoresistance to pyrazinamide, *n* = 3; streptomycin, *n* = 1; isoniazid, *n* = 1; ethambutol and isoniazid, *n* = 1). The remaining patients were classified as suspected CNS TB cases based on the clinical history and presentation, epidemiological risk factors, results of an interferon-gamma release assay (e.g., QuantiFERON^®^-TB Gold In-Tube test) or tuberculin skin test (Supplemental Table 2), radiologic findings, and CSF profile that warranted initiation of anti-TB treatment. Applying a uniform case definition used to categorize patients with CNS TB for research, 14 patients (26%) were classified as definite CNS TB, 23 (43%) as probable CNS TB, and 14 (26%) as possible CNS TB. Three patients could not be classified due to lack of CSF data. Among the 37 patients with probable or possible CNS TB, 13 (35%) had a potential alternative diagnosis but were empirically treated for CNS TB. All 3 cases of pyrazinamide monoresistance were microbiologically-confirmed *M. bovis* infection (mean age 37 years, none living with HIV). One patient with *M. bovis* infection died, while one survived with cognitive deficits and functional impairment.

Of 54 patients, 48 (89%) commenced a standard TB treatment regimen consisting of rifampin, isoniazid, pyrazinamide, and ethambutol (RHZE). Five patients received a liver-friendly regimen due to co-occurring conditions, and one patient died before initiating treatment. Of the 48 patients who began RHZE, 15 (31%) required at least one modification to their regimen because of adverse effects, such as hepatotoxicity and allergic reactions, or based on susceptibility data from positive cultures. Alternative regimens included moxifloxacin (*n* = 13), levofloxacin (*n* = 3), linezolid (*n* = 3), and streptomycin (*n* = 2). Among survivors, the mean duration of treatment for CNS TB was 11.5 months (SD 3.1 months). Adjunctive corticosteroids were administered during the initial phase of treatment in 37 (69%) patients.

During a mean follow-up of 10.2 months, 12 patients died, corresponding to a cumulative mortality of 22%. For those who died, the mean interval from initiation of anti-TB therapy to death was 4.8 months (SD 6.0 months, range 1 to 20 months). Of the 42 survivors, 17 (40%) demonstrated functional impairment, as indicated by a Katz ADL Index score of 4 or lower, and 16 (38%) exhibited moderate to severe cognitive limitations.

Individuals who died were significantly more likely to have a history of hypertension, diabetes mellitus, or end-stage renal disease, as well as altered mental status at presentation and need for intubation (Table 2). Functional impairment was associated with older age, public insurance, altered mental status at presentation, and hydrocephalus (Table 2). Survivors with moderate to severe cognitive dysfunction were significantly more likely to reside in neighborhoods with greater deprivation according to the ADI, to be altered or require intubation at presentation, and to have hydrocephalus on imaging (Table 2).

**Table 2:** Clinical and sociodemographic factors by mortality, functional impairment, and cognitive impairment

Characteristic, *n* (%) unless noted	Mortality (*N*=54)			Functional Impairment (*N*=42)			Cognitive Impairment (*N*=42		
	Dead	Alive	*p*-value	Yes	No	*p*-value	Yes	No	*p*-value
Age (mean), years	58	44	0.075	54	37	0.015	51	40	0.15
Sex at birth									
Male	7 (13)	23 (43)	1.00	9 (21)	14 (33)	0.85	9 (21)	14 (33)	0.88
Female	5 (9)	19 (35)		8 (19)	11 (26)		7 (17)	12 (29)	
Race and Ethnicity									
Non-Hispanic Asian	5 (9)	17 (31)	1.00	6 (14)	11 (26)	0.18	7 (17)	10 (24)	0.92
Non-Hispanic White	3 (6)	12 (22)		3 (7)	9 (21)		4 (10)	8 (19)	
Other	4 (7)	13 (24)		8 (19)	5 (12)		5 (12)	8 (19)	
US-Born									
No	7 (13)	27 (50)	0.80	11 (26)	16 (38)	0.60	8 (19)	19 (45)	0.23
Yes	5 (9)	14 (26)		5 (12)	9 (21)		7 (17)	7 (17)	
Unknown	0 (0)	1 (2)		1 (2)	0 (0)		1 (2)	0 (0)	
Used a Translator									
Yes	4 (7)	16 (30)	0.52	8 (19)	8 (19)	0.32	6 (14)	10 (24)	0.95
No	8 (15)	26 (48)		9 (21)	17 (40)		10 (24)	16 (38)	
Primary Insurance									
Public	9 (17)	27 (50)	0.29	16 (38)	11 (26)	0.001	12 (29)	15 (36)	0.073
Private	1 (2)	11 (20)		0 (0)	11 (26)		2 (5)	9 (21)	
Uninsured	0 (0)	2 (4)		0 (0)	2 (5)		0 (0)	2 (5)	
Unknown	2 (4)	2 (4)		1 (2)	1 (2)		2 (5)	0 (0)	
Housing Status									
Unhoused or unstably housed	0 (0)	2 (4)	0.60	0 (0)	2 (5)	0.35	0 (0)	2 (5)	0.38
Stably housed	12 (22)	40 (74)		17 (40)	23 (55)		16 (38)	24 (57)	
Area Disadvantage Index (decile)									
≤ 7	10 (19)	27 (50)	0.19	9 (21)	18 (43)	0.21	7 (17)	20 (48)	0.029
≥ 8	2 (4)	15 (28)		8 (19)	7 (17)		9 (21)	6 (14)	
Living with HIV Infection									
Yes	1 (2)	5 (9)	0.82	1 (2)	4 (10)	0.82	1 (2)	4 (10)	0.21
No	10 (19)	35 (65)		15 (36)	20 (48)		13 (31)	22 (52)	
Unknown	1 (2)	2 (4)		1 (2)	1 (2)		2 (5)	0 (0)	
History of hypertension									
Yes	8 (15)	13 (24)	0.030	7 (17)	6 (14)	0.24	7 (17)	6 (14)	0.16
No	4 (7)	29 (54)		10 (24)	19 (45)		9 (21)	20 (48)	
History of diabetes									
Yes	6 (11)	7 (13)	0.014	3 (7)	4 (10)	0.66	3 (7)	4 (10)	0.51
No	5 (9)	34 (63)		13 (31)	21 (50)		12 (29)	22 (52)	
Unknown	1 (2)	1 (2)		1 (2)	0 (0)		1 (2)	0 (0)	
History of end-stage renal disease									
Yes	2 (4)	0 (0)	0.046		---*			---*	
No	10 (19)	42 (78)							
Weight (mean)	132.1	134.2	0.91	143.59	127.58	0.34	131.34	136.04	0.78
Altered Mental Status									
Yes	10 (17)	20 (37)	0.040	13 (31)	7 (17)	0.005	14 (33)	6 (14)	<0.001
No	2 (4)	20 (37)		4 (10)	16 (38)		2 (5)	18 (43)	
Need for Intubation									
Yes	7 (13)	9 (17)	0.020	5 (12)	4 (10)	0.25	6 (14)	3 (7)	0.056
No	5 (9)	33 (61)		12 (29)	21 (50)		10 (24)	23 (55)	
Abnormal Lung Exam									
Yes	7 (13)	21 (39)	0.43	9 (21)	12 (29)	0.75	9 (21)	12 (29)	0.53
No	5 (9)	21 (39)		8 (19)	13 (31)		7 (17)	14 (33)	
Microbiological Confirmation									
Yes	4 (7)	10 (19)	0.37	3 (7)	7 (17)	0.35	4 (10)	6 (14)	0.59
No	8 (15)	32 (59)		14 (33)	18 (43)		12 (29)	20 (48)	
Hydrocephalus									
Yes	5 (9)	15 (28)	0.48	10 (24)	5 (12)	0.012	12 (29)	3 (7)	<0.001
No	7 (13)	27 (50)		7 (17)	20 (48)		4 (10)	23 (55)	
Tuberculoma/mass lesion									
Yes	5 (9)	13 (24)	0.36	4 (10)	9 (21)	0.31	3 (7)	10 (24)	0.16
No	7 (13)	29 (54)		13 (31)	16 (38)		13 (31)	16 (38)	
Steroid Use									
Yes	8 (15)	29 (54)	0.56	14 (33)	15 (36)	0.12	12 (29)	17 (40)	0.38
No	3 (6)	13 (24)		3 (7)	10 (24)		4 (10)	9 (21)	
Alcohol Use									
4 or more drinks per week	2 (4)	6 (11)	0.44	3 (7)	3 (7)	0.50	1 (2)	5 (12)	0.44
3 or fewer drinks per week	10 (19)	29 (54)		10 (24)	19 (45)		11 (26)	18 (43)	
Missing	0 (0)	7 (13)		4 (10)	3 (7)		4 (10)	3 (7)	
Tobacco use (smoking)									
Current	2 (4)	6 (11)	0.21	0 (0)	6 (14)	0.14	0 (0)	6 (14)	0.14
Former	4 (7)	5 (9)		3 (7)	2 (5)		3 (7)	2 (5)	
Never	6 (11)	24 (44)		11 (26)	13 (31)		11 (26)	13 (31)	
Unknown	0 (0)	7 (13)		3 (7)	4 (10)		2 (5)	5 (12)	
Marijuana Use									
Yes	1 (2)	5 (9)	1.00	2 (5)	3 (7)	1.00	2 (5)	3 (7)	1.00
No	11 (20)	37 (69)		15 (36)	22 (39)		14 (33)	23 (55)	

*Unable to assess association with functional or cognitive impairment as no patients with ESRD survived

**Fig. 3:**
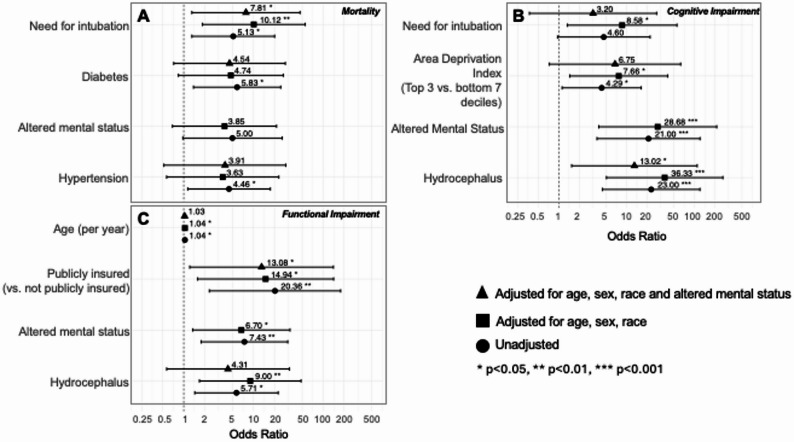
Clinical and social determinants of treatment outcomes in patients with CNS TB. Determinants of mortality (**A**), cognitive impairment (**B**) and functional impairment (**C**) are shown

In unadjusted logistic regression models, hypertension and diabetes mellitus were associated with higher odds of mortality [odds ratio (OR) 4.46, 95% CI 1.14–17.50, *p* = 0.027 for hypertension; OR 5.83, 95% CI 1.38–24.57, *p* = 0.016 for diabetes]. These associations were not significant after adjusting for age, sex, and race/ethnicity (Table 3; Fig. 3) or for age alone [adjusted OR (aOR) 3.63 for hypertension, 95% CI 0.57–23.14, *p* = 0.17; aOR 4.74 for diabetes, 95% CI 0.84–26.79, *p* = 0.08]. Both conditions remained significant predictors of mortality after adjustment for ADI status. The need for intubation at presentation was also associated with higher odds of mortality in both unadjusted and adjusted models (Table 3; Fig. 3).

**Table 3:** Clinical and Sociodemographic Factors Associated with Mortality, Functional Impairment, and Cognitive Impairment

	Unadjusted Model	Adjusted for age, sex, and race	Adjusted for age, sex, race, and AMS^1^	Adjusted for Area Deprivation Index
	OR (95% Confidence Interval)	OR 95% Confidence Interval)	OR 95% Confidence Interval)	OR 95% Confidence Interval)
Mortality				
History of hypertension	4.46 (1.14, 17.50)*	3.63 (0.569, 23.14)	3.91 (0.52, 29.17)	4.89 (1.20, 19.93)*
History of diabetes	5.83 (1.38, 24.57)*	4.74 (0.84, 26.79)	4.54 (0.72, 28.65)	5.50 (1.28, 23.55)*
Altered mental status	5.00 (0.97, 25.76)	3.85 (0.69, 21.44)	---	6.69 (1.22, 36.57)*
Need for intubation	5.13 (1.31, 20.08)*	10.12 (1.85, 55.41)**	7.81 (1.31, 46.55)*	7.54 (1.65, 34.35)**
Functional Impairment				
Age (per year)	1.04 (1.01, 1.07)*	1.04 (1.01, 1.08)*	1.03 (0.997, 1.07)	1.04 (1.01, 1.08)*
Publicly insured (vs. all other insurance status)	20.36 (2.33, 178.20)**	14.94 (1.58, 141.29)*	13.08 (1.22, 140.28)*	19.72 (2.13, 182.47)**
Altered mental status	7.43 (1.78, 31.04)**	6.70 (1.34, 33.45)*	---	6.97 (1.62, 30.06)**
Hydrocephalus	5.71 (1.44, 22.62)*	9.00 (1.68, 48.16)**	4.31 (0.57, 32.76)	5.21 (1.20, 22.57)*
Cognitive Impairment				
Area Deprivation Index (top 3 vs. bottom 7 deciles)	4.29 (1.12, 16.44)*	7.66 (1.46, 40.22)*	6.75 (0.73, 62.40)	---
Altered mental status	21.00 (3.66, 120.36)***	28.68 (3.88, 212.19)***	---	18.69 (3.16, 110.65)***
Need for intubation	4.60 (0.96, 22.16)	8.58 (1.34, 55.05)*	3.20 (0.37, 27.61)	3.77 (0.73, 19.59)
Hydrocephalus	23.00 (4.41, 119.96)***	36.33 (5.05, 261.32)***	13.02 (1.55, 109.42)*	18.77 (3.44, 102.24)***

AMS, altered mental status

^1^Two patients for whom AMS could not be determined were excluded from the model

* *p* < 0.05; ** *p* < 0.01; *** *p* < 0.001

Older age (OR 1.04 per year of age, 95% CI 1.01–1.07, *p* = 0.023), altered mental status (OR 7.43, 95% CI 1.78–31.04, *p* = 0.006), and hydrocephalus (OR 5.71, 95% CI 1.44–22.62, *p* = 0.013) were associated with higher odds of functional impairment in both unadjusted models and models adjusted for demographics. Public insurance was also linked to higher odds of functional impairment across all models (OR 20.36, 95% CI 2.33–178.20, *p* = 0.006; aOR 14.94, 95% CI 1.58-141.29, *p* = 0.018 adjusted for demographics) (Table 3; Fig. 3). After adjusting for demographics and ADI, older age, altered mental status, and hydrocephalus remained significantly associated with functional impairment. (Table 3; Fig. 3).

ADI was associated with higher odds of cognitive impairment in an unadjusted model (OR 4.29, 95% CI 1.12–16.44, *p* = 0.034) and after adjusting for age, sex, and race (aOR 7.66, 95% CI 1.46–40.22, *p* = 0.016). However, this association was not significant after adding altered mental status to the model (aOR 6.75, 95% CI 0.73–62.40, *p* = 0.09). Both altered mental status and hydrocephalus were also associated with higher odds of cognitive impairment in unadjusted and adjusted models (Table 3). After excluding the 3 patients who were unable to be unclassified by the uniform case definition due to lack of CSF data, no change was observed in the factors significantly associated with mortality, functional impairment, or cognitive impairment. After further exclusion of the 14 patients classified as possible cases, the association of hydrocephalus with functional impairment was no longer statistically significant (OR 2.88, 95% CI 0.60-13.75, *p* = 0.19).

## Discussion

In this study, patients with CNS TB cared for over a 10-year period in a high-income setting had a cumulative mortality of 22%. This finding is in line with previously reported mortality rates of 20% to over 40%, with higher rates observed in people living with HIV and those with drug-resistant TB [3, 28, 29]. Furthermore, 40% of patients who survived had significant limitations in day-to-day function, and a similar proportion suffered from moderate to severe cognitive impairment. This prevalence of disability is comparable to other studies that have reported treatment outcomes in CNS TB, again predominantly in low resource, high TB burden settings [10, 13–15, 18, 30]. The effect of this degree of disability on quality of life and productivity underscores the potential economic impact of CNS TB at the individual and societal level, especially given the overall young age of patients affected by CNS TB.

Our cohort comprised a typical U.S. patient population affected by CNS TB: foreign-born, first-generation immigrants, many of whom reside in areas of high deprivation and social vulnerability and may face language and other socioeconomic barriers. We captured information about social determinants of health based on insurance, housing status, need for a translator, and area-level socioeconomic disadvantage using the ADI. Although poverty is a well-established risk factor for TB and for worse outcomes associated with TB, none of these factors was independently associated with mortality in CNS TB [31]. This may have been due to the small sample size, or the lack of substantial variability in socioeconomic measures (e.g., 96% of the cohort was housed). Furthermore, access to urgent health care through local safety net hospitals and to specialty care through the Department of Public Health’s TB clinic may have mitigated the impact of socioeconomic status on mortality. However, patients with public insurance were more likely to be living with functional impairment after surviving CNS TB. Similarly, patients residing in areas with greatest deprivation, as measured by the ADI, were more likely to have moderate to severe cognitive limitations than patients in less deprived neighborhoods. These exploratory findings suggest that socioeconomic factors may influence, in part, the likelihood of surviving CNS TB with residual disability, which has implications on quality of life, participation in society, and financial independence. The associations between public insurance, ADI, and treatment outcomes, however, should be interpreted cautiously given the wide confidence intervals around point estimates. Competing responsibilities and barriers to seeking initial care and obtaining follow-up care (e.g., lack of transportation, housing instability, caregiving duties, inflexible employment, low health literacy) and differential access to rehabilitation and other post-acute services are plausible pathways by which patients with lower socioeconomic status may be at risk for worse long-term outcomes in CNS TB. More nuanced data on social determinants of health, including measures of economic stability and social support networks, combined with a larger sample size will be critical to elucidating the impact of social determinants of health on outcomes and developing targeted interventions for patients with CNS TB.

Diabetes mellitus, which was common in the cohort, was independently associated with a higher risk of mortality. These results extend findings from studies performed in a wide range of settings indicating that comorbid diabetes mellitus increases the odds of poor treatment outcomes and death from pulmonary TB to patients with CNS TB [32]. People with diabetes mellitus are at higher risk of not only developing active TB infection but also of more severe disease. TB relapse, treatment failure, and drug-resistance [33]. A U.S. study of patients with CNS TB in Florida found the risk of death associated with co-morbid diabetes mellitus was higher than HIV co-infection [29]. In light of rapidly rising rates of diabetes in the U.S. and in LMIC where the burden of TB is high, enhanced bidirectional screening in high-risk populations and optimization of management of diabetes in patients with and at risk for TB may improve outcomes in this high-risk population. In contrast to mortality, diabetes was not associated with worse functional or cognitive outcomes. This discrepancy may have been a limitation of the smaller sample size of survivors, as nearly half of patients with diabetes died. In addition, selection bias in the survivor population could explain the lack of an association, as people with diabetes mellitus who survived may have had less severe disease and, thus, average or better outcomes.

Although the data implicating hypertension as a negative prognostic factor is more limited than for diabetes mellitus, a few studies have demonstrated an association between co-occurring hypertension and increased mortality in pulmonary TB [34, 35]. Like diabetes mellitus, hypertension was a risk factor for death in the present study, although the relationship was attenuated after adjusting for age, sex, and race, and for age alone. This suggests that older age, a known risk factor for hypertension, may have been partially driving the link between hypertension and mortality. Alternatively, older age may be a proxy for the duration or poor control of hypertension, which could explain the observed association between hypertension and mortality in patients with CNS TB.

HIV and TB co-infection is known to significantly increase the risk of mortality, including in patients with CNS TB, treatment failure, and relapse of TB. The prevalence of HIV infection was higher in the cohort than in the general population, with patients being approximately 32-fold more likely to be living with HIV compared with the general population of California [36]. We did not observe a significant difference in outcome by HIV status, which may have been related to the modest sample size. In addition, all but one of the patients living with HIV were on ART, which may explain similar outcomes among those with and without HIV. No health-related behaviors, including alcohol use or cigarette smoking, both of which have been linked to poorer outcomes in pulmonary TB in predominantly LMIC settings, were associated with worse clinical outcomes [37].

Altered mental status at presentation was associated with increased mortality and higher risk of functional and cognitive impairment. Previous studies have demonstrated that the stage of disease at presentation, which indicates severity of infection, is among the strongest predictors of mortality [38]. We were not able to systematically assign the stage of disease at presentation using the modified British Medical Research Council (BMRC) grading system as the Glasgow Coma Score (GCS), a key component of the BMRC grade, was infrequently assessed or documented in clinical notes. However, altered mental status at presentation, which is reflected in the GCS, may serve as a proxy for disease severity. The need for intubation and presence of hydrocephalus, which can similarly be viewed as indicators of disease severity that correlate, and may be collinear, with altered mental status, were also associated with mortality and functional or cognitive impairment.

Only one-quarter of patients in this study had microbiologically or molecularly confirmed disease [7]. Given the challenge of diagnosing TBM, a subset of patients, despite receiving a full course of anti-TB therapy, may not have had TBM. Further, about one-third of patients did not receive corticosteroids, which have been shown to improve outcomes in TBM, potentially reflecting differences in management of CNS TB in the face of uncertainty around the diagnosis. No significant association was found between corticosteroid use and outcomes. Among cases of microbiologically confirmed TBM, none had multi-drug-resistant TB infection. Although the cohort included only a small number of *M. bovis *infections, we did not observe a statistically significant association of *M. bovis* with mortality or any clinical outcome, despite reports of more severe presentations and worse overall prognosis in patients with *M. bovis*infection [39, 40].

This study has several limitations. First, the modest sample size constrained statistical power to detect associations between variables and clinical outcomes, as reflected by wide confidence intervals indicating considerable uncertainty in the magnitude of observed effects. Second, cognitive and functional status at follow-up was ascertained from clinical documentation rather than prospectively collected validated measures, and was inconsistently available across patients, potentially introducing misclassification bias. Third, two sources of selection bias may have affected our results. Referral bias arising from the tertiary center setting at UCSF means our sample likely overrepresents complex or severe cases. In addition, differential loss to follow-up poses a threat to the validity of our findings. Patients with better clinical outcomes may have been lost to follow-up or transitioned care to primary care settings, while those with worse clinical outcomes or greater socioeconomic barriers may have been unable to return for follow-up. Patients with fewer clinical encounters may also have had less documentation of cognitive and functional outcomes, further contributing to outcome ascertainment bias. Fourth, residual confounding is likely given the constraints of medical record data. Key social determinants of health, including educational attainment, income, employment and social support, were incompletely captured. Data on health-related behaviors may have been outdated or reflected status at follow-up rather than preceding the CNS TB diagnosis, hampering our ability to account for their confounding effects. While these findings may ultimately be most applicable to comparable tertiary referral centers in urban settings, they provide a foundation for characterizing the burden of CNS TB in the U.S. and other high-income countries. Strengths of the study include the focus on a U.S. population in a high-resource setting and in-depth review of medical records to characterize clinical outcomes that are not readily captured by administrative or billing codes.

In this high-income setting in the U.S., cumulative mortality and rates of disability in patients with CNS TB mirrored treatment outcomes in studies from LMIC. In addition to older age, comorbid conditions like diabetes mellitus, and markers of more severe disease at presentation, having public insurance and greater neighborhood-level social disadvantage were also associated with worse clinical outcomes. Given key limitations of the study, including the modest sample size, retrospective design, and reliance on medical records, these exploratory findings should be viewed as hypothesis-generating and require confirmation in future work that further characterizes the impact of psychosocial and socioeconomic factors on treatment outcomes of CNS TB. If validated in larger studies with prospectively collected data, a multi-pronged approach to improve outcomes in CNS TB should consider adverse social determinants of health that disproportionately impact people at risk for TB and targeted public health strategies to promote earlier presentation, recognition, diagnosis, and treatment of this devastating form of TB among socially and economically marginalized populations.

## Supplementary Information

Below is the link to the electronic supplementary material.

Supplementary Material 1

## Data Availability

The datasets used and analyzed for the study are available upon reasonable request by qualified investigators following approval by the corresponding author.

## Abbreviations

ADI: Area Deprivation Index
ADL: Activity of daily living
AFB: Acid-fast bacilli
ART: Antiretroviral therapy
BMRC: British Medical Research Council
CNS: Central nervous system
CSF: Cerebrospinal fluid
GCS: GLASGOW Coma Score
ICD: International Classification of Diseases
LMIC: Low and middle-income countries
OR: Odds ratio
PWH: People living with HIV
SD: Standard deviation
TB: Tuberculosis
