# Supplementary material for: Clinical and social determinants of treatment outcomes in central nervous system tuberculosis in a high-resource setting: a retrospective cohort study
Source: BMC Infect Dis. 2026 Apr 28;26:1147. doi: 10.1186/s12879-026-13439-8 (PMC13267490; doi:10.1186/s12879-026-13439-8)
Supplement: Supplementary file 1 — Supplementary Material 1 [file 12879_2026_13439_MOESM1_ESM.docx]

Supplemental Table 1. Clinical Signs and Symptoms at Presentation

| Characteristic, n (%) | | | | |
| --- | --- | --- | --- | --- |
| Headache |  | Fever |  |  |
| Present | 33 (61) | Present | 39 (72) | |
| Absent | 15 (28) | Absent | 14 (26) | |
| Unknown / Unable to Assess | 6 (11) | Unknown / Unable to Assess | 1 (2) | |
| Visual Changes |  | Night Sweats |  | |
| Present | 15 (28) | Present | 12 (22) | |
| Absent | 30 (56) | Absent | 40 (74) | |
| Unknown / Unable to Assess | 9 (17) | Unknown / Unable to Assess | 2 (4) | |
| Photophobia |  | Weight Loss |  | |
| Present | 7 (13) | Present | 24 (44) | |
| Absent | 39 (72) | Absent | 28 (52) | |
| Unknown / Unable to Assess | 8 (15) | Unknown / Unable to Assess | 2 (4) | |
| Altered Mental Status |  | Nausea/Vomiting |  | |
| Present | 30 (56) | Present | 25 (46) | |
| Absent | 22 (40) | Absent | 26 (48) | |
| Unknown / Unable to Assess | 2 (4) | Unknown / Unable to Assess | 3 (6) | |
| Focal Weakness |  | Requiring intubation |  | |
| Present | 38 (70) | Present | 16 (30) | |
| Absent | 16 (30) | Absent | 38 (70) | |
| Seizures |  | Rash |  | |
| Present | 15 (28) | Present | 5 (9) | |
| Absent | 35 (65) | Absent | 45 (83) | |
| Unknown / Unable to Assess | 4 (7) | Unknown / Unable to Assess | 4 (7) | |
| Back Pain |  | Lymphadenopathy |  | |
| Present | 9 (17) | Present | 9 (17) | |
| Absent | 45 (83) | Absent | 45 (83) | |
| Cranial nerve palsy |  | Abnormal lung exam |  | |
| Present | 15 (28) | Present | 28 (52) | |
| Absent | 39 (72) | Absent | 26 (48) | |
| Other focal neurologic deficit |  | Abnormal abdominal exam | | |
| Present | 21 (39) | Present | 14 (25) | |
| Absent | 33 (61) | Absent | 40 (75) | |
| Nuchal rigidity |  | Hearing Loss |  | |
| Present | 10 (19) | Present | 5 (9) | |
| Absent | 44 (81) | Absent | 49 (91) | |
| Cough |  |  |  |  |
| Present | 26 (48) |  |  |  |
| Absent | 26 (48) |  |  |  |
| Unknown / Unable to Assess | 2 (4) |  |  |  |

Supplemental Table 2. Results of QuantiFERON Gold and tuberculin skin testing

| Test Results, n (%) | All  Patients | Microbiologically  confirmed | Not microbiologically  confirmed |
| --- | --- | --- | --- |
|  | (N=54) | (N=14) | (N=40) |
| QuantiFERON Gold |  |  |  |
| Positive | 20 (37) | 6 (43) | 14 (35) |
| Negative | 14 (26) | 1 (7) | 13 (33) |
| Indeterminate | 3 (6) | 1 (7) | 2 (5) |
| Not Available | 17 (32) | 6 (43) | 11 (28) |
| Tuberculin Skin Test |  |  |  |
| Positive | 15 (28) | 5 (36) | 10 (25) |
| Negative | 15 (28) | 2 (14) | 13 (33) |
| Not Available | 24 (44) | 7 (50) | 17 (43) |
